# Computational Electromagnetic Analysis of Deformed Nanowires Using the Multilevel Fast Multipole Algorithm

**DOI:** 10.1038/srep08469

**Published:** 2015-02-16

**Authors:** Akif Yılmaz, Barışcan Karaosmanoğlu, Özgür Ergül

**Affiliations:** 1 Department of Electrical and Electronics Engineering, Middle East Technical University, Ankara, Turkey

## Abstract

We consider computational analysis of deformed nanowires and their arrays using a full-wave simulation environment based on integral-equation formulations and the multilevel fast multipole algorithm (MLFMA). Without requiring any periodicity assumptions, MLFMA allows for fast and accurate simulations of complex nanowire structures with three-dimensional geometries and random deformations. We present the results of hundreds of simulations, where deformed nanowires are considered as isolated, as well as in array configurations, and their scattering characteristics are compared to those of non-deformed ones. Based on the simulation results, we rigorously investigate common effects of deformations on scattering properties of nanowires and identify strong field enhancements in forward-scattering directions.

Nanowires are popular structures in many application areas of electronics and optics^1^,^2^,^3^,^4^,^5^,^6^,^7^,^8^,^9^,^10^,^11^,^12^,^13^,^14^,^15^. Among diverse useful applications, they are commonly employed for constructing metamaterials at optical frequencies^4^,^5^,^9^. While a periodical arrangement of nanowires do not induce an effective double negativity (hence a negative refractive index), they lead to negative refraction that can be used for super-resolution imaging^9^ and for similar applications requiring low loss. Being manufactured easier than other structures, nanowires will remain as one of the most favourite building blocks of optical metamaterials, where they can be combined with other structures^10^,^12^ for desired responses, as practiced many times at lower frequencies^16^,^17^.

In many practical applications, nanowires must be in “perfect” shapes with non-varying cross sections along their longer dimensions, while geometric deformations are usually considered as undesired factors that may reduce the efficiency of metamaterials. Along this direction, numerical and computational analysis of nanowires have been limited to such ideal cases, leading to deviations when metamaterials involving nanowires are realised in real life and investigated in measurement setups. Despite recent advances in manufacturing nano structures, enabling the production of arrays of nanowires with almost perfect shapes, deformations and imperfections are unavoidable. This paper is one of the first attempts, to the best of Authors' knowledge, to systematically investigate such deformations and their effects on the optical properties of nanowires. We present the results of hundreds of simulations, where deformed nanowires are considered as isolated, as well as in array configurations, and their scattering characteristics are compared to those of non-deformed (perfect) ones.

With minimum initial assumptions, we consider scattering problems involving nanowires with random deformations. For thorough analysis, we consider various random distributions, maximum deformation limits, excitations, wire lengths, cross section shapes, and frequencies. Some of these parameters, such as the frequency, are omitted in the results of this paper as they do not directly affect the conclusions drawn from these numerical experiments. All problems are solved rigorously using the multilevel fast multipole algorithm (MLFMA)^18^,^19^ that allows for three-dimensional modelling of complex objects with arbitrary geometries^20^,^21^,^22^,^23^,^24^. Surface integral equations, which are discretized with the Rao-Wilton-Glisson (RWG) functions^25^, are used to formulate electromagnetics problems. Thanks to the low-complexity of MLFMA, hundreds of simulations become feasible without sacrificing the accuracy of results, allowing for statistically meaningful analysis of deformations. Based on the simulation results, we identify some common effects of deformations on scattering properties of nanowires, in contrast to unique scattering characteristics of perfect nanowires.

Based on the background information above, main motivations of this work can be emphasised as follows:

- Systematically investigating the effects of deformations on scattering properties of nanowires, hence providing more realistic simulations of real-life scenarios involving these important structures.
- Performing hundreds of simulations using a powerful three-dimensional solver such that statistically meaningful analysis of random deformations becomes possible.
- Characterising dependencies of scattering properties of nanowires at critical angles, such as backscattering and forward-scattering directions, to the severity of deformations.

While the main purpose of this work is to provide realistic data to support experimental analysis of nanowires, we also present a super-linear enhancement in their forward-scattered abilities via deformations, which may have practical values in various applications, such as light trapping and imaging.

## Computational Analysis of Nanowires Using MLFMA

We consider far-zone scattering from metallic nanowires, which are modelled as perfectly conducting. Such problems can be formulated with the electric-field integral equation (EFIE), the magnetic-field integral equation (MFIE), and the combined-field integral equation (CFIE) that is a convex combination of EFIE and MFIE. Even though MFIE and CFIE are known to be ideal formulations for quick convergence of iterative solutions, they lead to significantly inaccurate results for nanowires, as shown below. Therefore, EFIE is preferred for rigorous analysis of perfect and deformed nanowires. Discretizations of integral equations via the RWG functions lead to *N* × *N* dense matrix equations, where *N* is the number of unknowns. The resulting matrix equations are solved iteratively, where the matrix-vector multiplications are performed efficiently with MLFMA.

### Simulation Environment Based on MLFMA

MLFMA is based on the factorization and diagonalization of the Green's function via the addition theorem and the plane-wave expansion. A given object is recursively divided into subdomains (cubes) such that interactions between discretization elements (basis and testing functions) can be performed in a group-by-group manner. Based on the recursive clustering of the object, a tree structure is constructed by considering only nonempty subdomains. Then, aggregation, translation, and disaggregation stages are performed consecutively to compute electromagnetic interactions. Each aggregation stage starts with the coefficients of basis functions provided by the iterative core algorithm, e.g., GMRES, to compute radiated fields of subdomains at the lowest level, and continues to higher levels via phase shifts and interpolations. Translations are performed between far-zone subdomains, which are defined in accordance with the assumed buffering scheme, for translating radiated fields into incoming plane waves. Finally, disaggregation operations are executed for computing total incoming fields at all subdomain centres, from the top to the bottom of the tree structure, where they are received by testing functions.

MLFMA is well known in the literature^26^,^27^, and it can easily be applied to perfectly conducting nanowires without modifying standard implementations. Using MLFMA, nanowires and their arrays can be modelled as three-dimensional objects, without infinity, periodicity, and similarity assumptions. For a problem discretized with *N* unknowns, each matrix-vector multiplication can be performed with *O*(*N* log *N*) computational complexity. On the other hand, error parameters of MLFMA must be selected carefully for accurate analysis of structures, including nanowires. Based on diverse convergence tests, we use the following setup in the simulations of nanowires presented in this paper.

- Far-zone interactions are determined using a one-box-buffer scheme, where touching (intersecting at a surface, at a line, or at a point) subdomains are considered to be in the near zone of each other. At the lowest level, interactions between basis and testing functions that are located in near-zone subdomains are performed directly via integrations on the supports of the functions. As commonly practiced, singularity extractions are employed to reduce the number of integration points for a given error threshold, which is selected as 1% in our simulations. These near-field interactions, which are calculated directly before iterative solutions, are stored in memory to be used multiple times during iterations.
- The numbers of harmonics that are used to compute far-zone interactions are determined using a refined excess bandwidth formula^28^. Similar to near-zone interactions, we set the maximum error to 1%, which corresponds to the error in the worst case, i.e., when both basis and testing functions are located at the corners of the associated subdomains. To maintain the same error level through the tree structure, we use the Lagrange interpolation with 4 × 4 stencils. Such a local interpolation scheme leads to less than 0.1% error, provided that the poles are handled carefully^27^.
- In iterative solutions, we use 0.1% residual error as the target for deciding a convergence. Such an error threshold can be difficult to satisfy for EFIE. Therefore, we use algebraic preconditioners to accelerate the convergence and to keep the number of iterations in reasonable limits.

Following the rules listed above, coefficients for expanding the electric current induced on nanowires are obtained with maximum 1% error. Numerical errors in far-zone scattered fields are usually lower due to smoothing effects of radiation integrals. Unfortunately, the type of the integral equation and its discretization can be major factors on the accuracy of simulations, especially when other error sources are suppressed.

### Three-Dimensional Modelling of Nanowires and Their Excitations

At this stage, it is convenient to describe the nanowire models and their excitations in the simulations. A perfect nanowire (without deformations) is defined as a 0.5 × 0.5 × 25 *μ*m rectangular prism with the longer dimension be oriented in the *z* direction. In addition to scenarios involving single nanowires, we consider periodic arrangements, such as 5 × 5 and 10 × 10 arrays, where nanowires are placed regularly with 1 *μ*m periods in the *x* and *y* directions, without any shifts in the *z* direction. Structures are excited either by a plane wave or a Hertzian dipole. The plane wave is propagating in the *z* direction with the electric field polarised in the *x* direction. The dipole is oriented in the *x* direction and placed symmetrically at 5 *μ*m above the given structure. In all results presented in this paper, the frequency is fixed to 50 THz. Considering that far-zone scattering properties are investigated, nanowires with given dimensions and that are made of highly conducting materials, such as silver, can be modelled as perfectly conducting. Analysis at higher frequencies, however, would require plasmonic modelling by adding magnetic currents and using penetrable formulations^29^.

**Figure 1:**
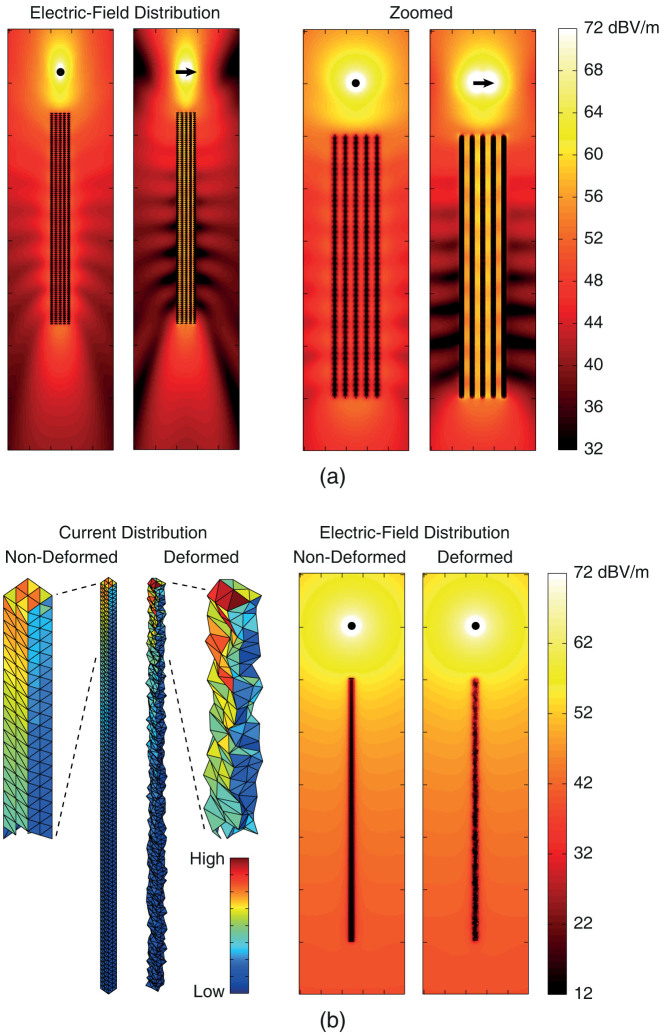
Examples of simulations of nanowires. (a) Near-zone electric-field intensity in the vicinity of a 5 × 5 nanowire array illuminated by a Hertzian dipole at 50 THz. (b) Electric current density induced on and near-zone electric-field intensity in the vicinity of a deformed/non-deformed nanowire illuminated by a Hertzian dipole at 50 THz. Each nanowire has dimensions of 0.5 × 0.5 × 25 *μ*m, with [−100, 100] nm shifts in the lateral directions for the deformed nanowire.

When formulated with EFIE, surfaces of nanowires are discretized with 250–350 nm (around *λ*/20) triangles, leading to 2400 unknowns per nanowire. This selection of the mesh size is based on many convergence analysis, some of which are presented in this paper. Apart from mesh convergences, accuracy of simulations are further verified by testing the equivalence principle. For perfectly conducting models, the equivalence theorem requires zero electric and magnetic fields inside objects. Hence, by computing fields in the vicinity of structures, one can measure the accuracy of solutions. As an example, Fig. 1(a) depicts the near-zone electric-field intensity in the vicinity of a 5 × 5 nanowire array illuminated by a Hertzian dipole. Both two planes (*z*-*x* and *z*-*y*) containing the dipole are considered, while the location and orientation of the dipole are clearly described in the plots. In Fig. 1(a), particularly in the zoomed plots, nanowires are visible as black lines due to vanishingly small fields inside them. Specifically, field values inside nanowires are 30–40 dB less than those between them, verifying the high accuracy of solutions. We also note that far-zone fields are usually obtained more accurately than near-zone fields, similar to their better accuracies compared to current distributions.

### Deformations of Nanowires

Nanowires can be deformed in many different ways. In order to investigate effects of various deformations, we consider alternative probability distribution functions (PDFs) and allow all discretization nodes moving freely in the lateral directions (*x* and *y* directions) in accordance with these random distributions. Four different distributions are of particular interest in this paper:

- 50% Uniform: Each node is moved with 50% probability, while each movement is uniformly distributed in a given range.
- 100% Uniform: Each node is definitely moved, while each movement is uniformly distributed in a given range.
- 50% Gaussian: Each node is moved with 50% probability, while each movement fits into a truncated Gaussian distribution in a given range.
- 100% Gaussian: Each node is definitely moved, while each movement fits into a truncated Gaussian distribution in a given range.

In addition to various PDFs, different ranges are considered by limiting the maximum deformation to ±5 nm, ±25 nm, ±50 nm, and ±100 nm. Hence, we consider very light (e.g., 50% Uniform/Gaussian with 5 nm maximum) irregularities, as well as quite aggressive (e.g., 100% Uniform/Gaussian with 100 nm maximum) deformations. An example to an aggressive deformation is depicted in Fig. 1(b), where the electric current distribution on the tip of a deformed nanowire is shown in comparison to that on the tip of the perfect one. We note that, even highly deformed nanowire samples may demonstrate basic scattering characteristics of wire structures if they are not investigated quantitatively. For example, as also depicted in Fig. 1(b), near-zone electric fields in the vicinities of perfect and deformed nanowires may look similar. On the other hand, one can actually identify significant changes in scattering values, especially in the forward-scattering directions, which cannot be neglected for an accurate analysis.

### Comparison of Surface Formulations

An important parameter for the accuracy of a nanowire simulation is the integral-equation formulation. It is already known in the literature that, despite they formulate the same physical problem using the Maxwell's equations, numerical solutions of integral equations may differ significantly in terms of accuracy and efficiency^29^,^30^,^31^,^32^,^33^. In the context of perfect conductors with closed surfaces, MFIE and CFIE provide fast iterative solutions since they are second-kind integral equations involving the identity operator. It is the same identity operator that reduces the accuracy of these formulations when they are discretized with low order functions, such as the RWG functions^34^. EFIE, however, is a first-kind integral equation, providing very accurate results at the cost of reduced efficiency due to increased numbers of iterations in iterative solutions. In many cases, inaccuracy of MFIE and CFIE may be in reasonable limits such that they are preferred against EFIE for fast solutions. In the case of nanowires, however, MFIE and CFIE are so inaccurate that their usage may indeed be much more inefficient in comparison to EFIE, for achieving similar accuracy levels.

**Figure 2:**
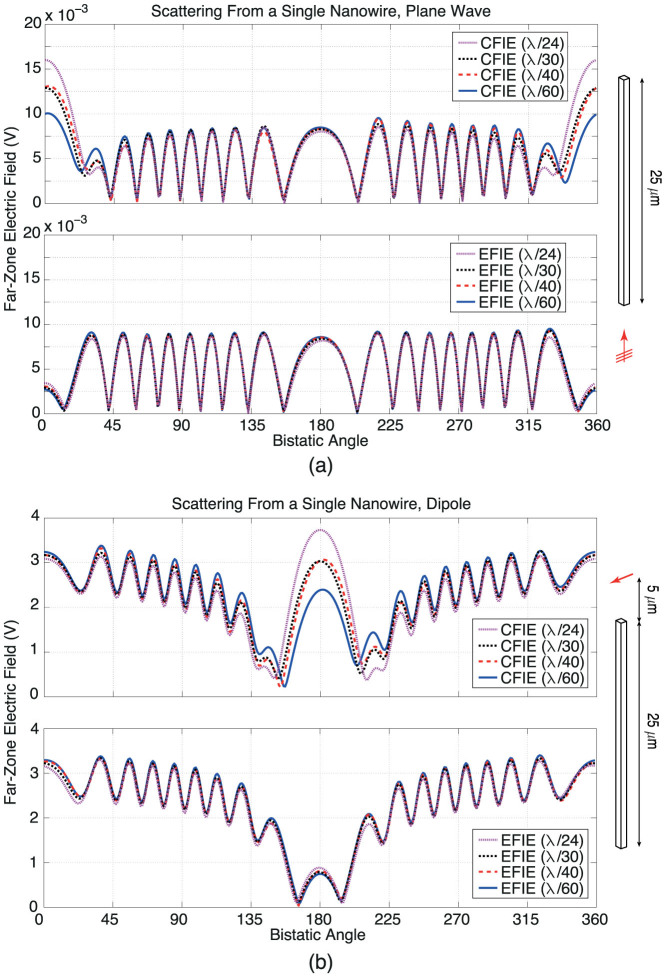
Far-zone electric field scattered from a single *z*-directed nanowire (0.5 × 0.5 × 25 *μ*m) illuminated by (a) plane wave (polarised in the *x* direction and propagating in the *z* direction) and (b) Hertzian dipole (oriented in the *x* direction and located symmetrically at 5 *μ*m above the nanowire) at 50 THz. The normalised electric-field intensity (V) is plotted on the *z*-*x* plane with respect to the bistatic angle *θ*.

Fig. 2 presents the results of numerical experiments involving a single perfect nanowire illuminated by a plane wave and a Hertzian dipole. Problems are formulated with EFIE and a balanced CFIE (EFIE/2 + MFIE/2), which are discretized with various triangulations involving *λ*/24–*λ*/60 triangles. By improving the discretization, we investigate the convergence of the far-zone electric field values at all bistatic angles on the *z*-*x* plane. As depicted in Fig. 2, CFIE results converge very slowly around the forward-scattering directions (0° for the plane-wave excitation and 180° for the dipole excitation), i.e., they change significantly when the mesh size changes. In the case of EFIE, however, a convergence is clearly observed with very consistent results for different mesh sizes. Although not shown in Fig. 2, it is also verified that, extremely fine discretizations of MFIE and CFIE can give accurate results that are similar to those obtained with EFIE.

Based on numerical experiments, such as depicted in Fig. 2, we conclude that EFIE is an inevitable choice for accurate simulations of nanowires, while MFIE and CFIE are not appropriate. It should be emphasised that this conclusion is made for discretizations with the RWG functions using a Galerkin scheme (using identical sets for basis and testing functions), while the accuracy of MFIE and/or CFIE can be improved significantly with alternative discretization schemes^35^,^36^.

## Numerical Results and Analysis

**Figure 3:**
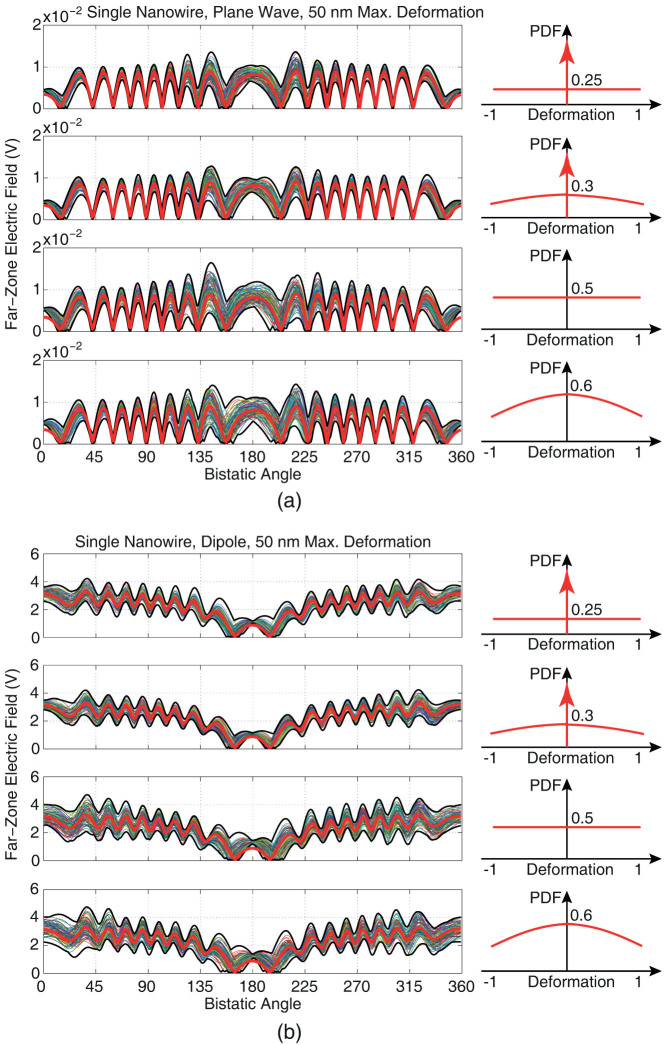
Far-zone electric field scattered from a single *z*-directed nanowire of length 25 *μ*m illuminated by (a) plane wave (polarised in the *x* direction and propagating in the *z* direction) and (b) Hertzian dipole (oriented in the *x* direction and located symmetrically at 5 *μ*m above the nanowire) at 50 THz. The original nanowire with 0.5 × 0.5 *μ*m cross section is deformed by moving each discretization node randomly in accordance with various probability distributions. All deformations are in the [−50, 50] nm range. Each plot contains 100 different trials/simulations, in addition to the results for the non-deformed nanowire (bold red lines).

Fig. 3 presents the results of scattering problems involving perfect and deformed single nanowires illuminated by (a) a plane wave and (b) a Hertzian dipole. We consider random deformation schemes with ±50 nm limits in accordance with four different PDFs as depicted on the right side of each plot. For each distribution scheme, 100 different nanowires are generated by randomly moving discretization nodes in accordance with the given PDF. Hence, each plot contains 100 different lines (trials), each representing the far-zone electric field on the *z*-*x* plane scattered from a deformed nanowire. In order to demonstrate the variation of scattered fields, minimum and maximum values are also shown with bold black lines. In addition, the results obtained for the non-deformed (perfect) nanowire are plotted with bold red lines. The following observations can be made:

- Deformations lead to significant deviations of scattered-field values from those obtained for the perfect nanowire. Both higher and lower values are observed, except in the forward-scattering directions, corresponding to 0° for the plane-wave excitation and 180° for the dipole excitation.
- In the forward-scattering directions, deformations always increase the value of the scattered field. Specifically, in those particular directions, the perfect nanowire gives unusually low scattered fields, which are easily amplified with deformations.

**Figure 4:**
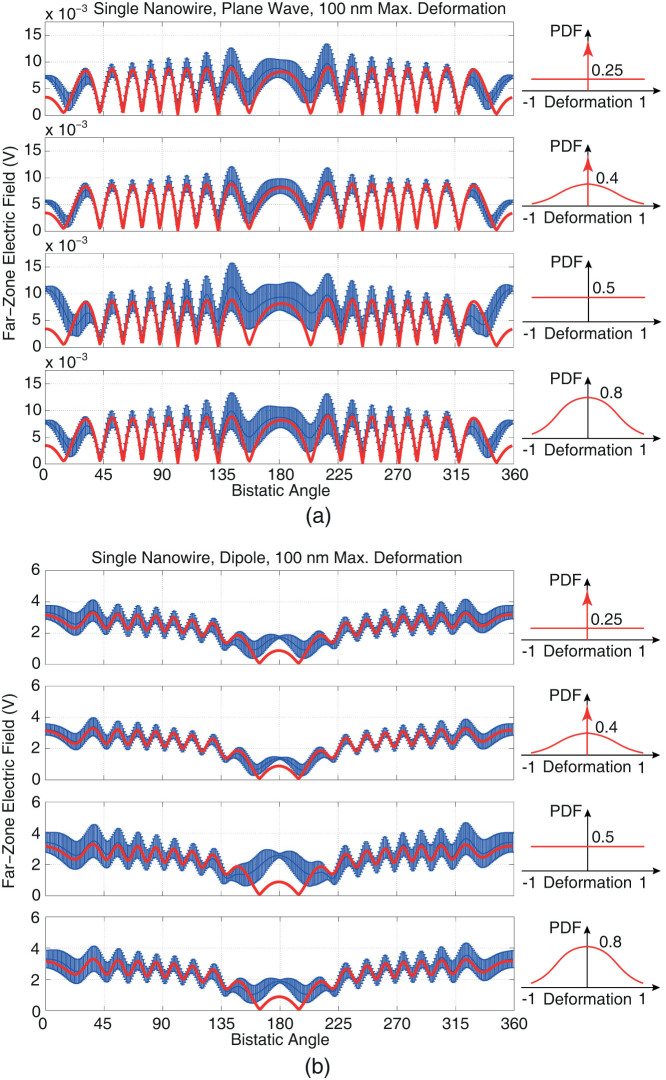
Far-zone electric field scattered from a single *z*-directed nanowire of length 25 *μ*m illuminated by (a) plane wave (polarised in the *x* direction and propagating in the *z* direction) and (b) Hertzian dipole (oriented in the *x* direction and located symmetrically at 5 *μ*m above the nanowire) at 50 THz. The original nanowire with 0.5 × 0.5 *μ*m cross section is deformed by moving each discretization node randomly in accordance with various probability distributions. All deformations are in the [−100, 100] nm range. Each plot contains the mean and standard deviation values obtained for 100 different trials/simulations, in addition to the results for the non-deformed nanowire (bold red lines).

Fig. 4 presents the results of a similar set of experiments involving single nanowires, where deformations are increased by making the limit ±100 nm. In these plots, mean and standard deviation values that are calculated by using 100 different trials are shown, in addition to the values obtained for the non-deformed nanowire (red bold lines), all again with respect to the bistatic angle on the *z*-*x* plane. The plots in Fig. 4 confirm previous conclusions that scattering values of deformed nanowires vary around the reference curves belonging the non-deformed one, except in the forward-scattering directions, where all deformations increase the scattering value. Since the forward-scattering is a quantity related to the electrical size of a structure, we further emphasise that deformations seem to increase the electrical size of the nanowire significantly, while its cross section is allowed to increase from 0.5 × 0.5 *μ*m to only 0.7 × 0.7 *μ*m.

**Figure 5:**
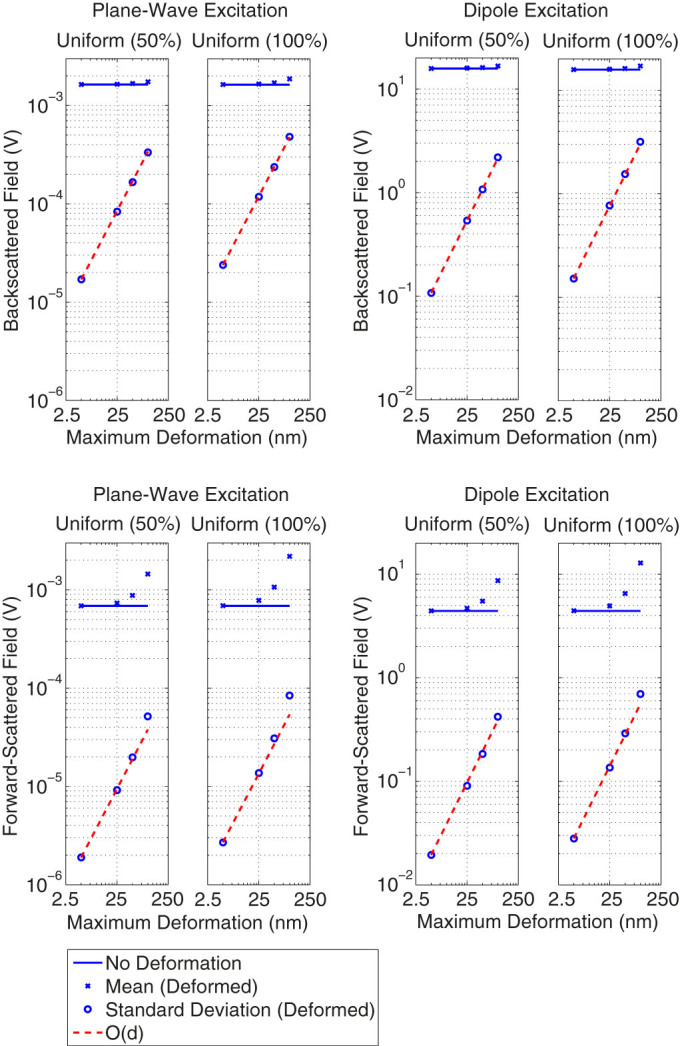
Backscattering and forward-scattering characteristics of deformed single nanowires based on 1600 different numerical experiments.

Next, for a rigorous analysis, we collect the results of a total of 1600 simulations in Fig. 5. We consider again plane wave and dipole excitations of single nanowires, two types of random distributions (Uniform 50% and Uniform 100%), and various limits for deformations, i.e., ±5 nm, ±25 nm, ±50 nm, and ±100 nm. This way, we investigate scattering characteristics of nanowires with respect to the intensity of the deformation. Each plot in Fig. 5 contains mean and standard deviation values (based on 100 trials) of scattered fields for deformed nanowires in contrast to the field value for the non-deformed one. For both types of excitations, the following observations can be made:

- The backscattered electric field, which is related to the physical cross section of a structure, slightly increases with the intensity of the deformation. The standard deviation values fit into an *O*(*d*) curve, where *d* represents the maximum deviation, verifying that deviations of discretization nodes are statistically consistent with the given PDFs.
- The forward-scattered electric field rapidly increases as the deformations become more intense. These are again consistent with previous results, verifying the increasing electrical size with deformations. The standard deviation is also large for aggressive deformations, even exceeding the *O*(*d*) curve, while it does not seem to be sufficiently large to generate many deformed designs with low forward-scattering values.

**Figure 6:**
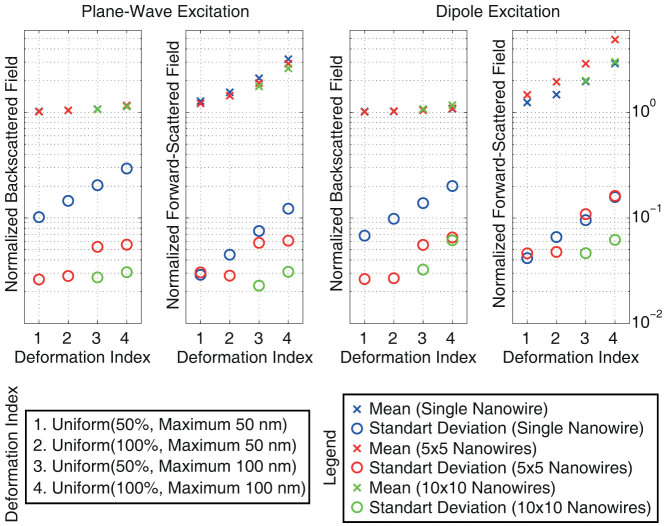
Backscattering and forward-scattering characteristics of deformed nanowires and their arrays. Each data point is obtained by using 100 simulations for a single nanowire, 20 simulations for a 5 × 5 array, and 10 simulations for a 10 × 10 array.

Fig. 6 presents an analysis involving arrays of nanowires with deformations. Four different deformation schemes are considered and applied to generate 100 different single nanowires and 20 different 5 × 5 arrays. In addition, aggressive deformations with ±100 nm limits are used to generate 10 different 10 × 10 arrays. The mean and standard deviation values obtained for the backscattered and forward-scattered electric field are plotted with respect to the deformation type, when all structures are excited with a plane wave and a Hertzian dipole. For comparing scattering characteristics of differently sized structures, field values are normalised by the reference values obtained for the corresponding non-deformed structures. Hence, a unity mean value corresponds to the scattering from a non-deformed structure. Our observations are as follows:

- In the backscattering direction, all values are close to unity as expected, due to relatively small increases in the physical cross sections of structures. Mutual couplings between nanowires seem to stabilise the backscattered electric field against deformations, since the standard deviation tends to decrease in array configurations.
- Similar to single nanowires, forward-scattered electric field of a deformed array tends to be significantly larger than the reference value of the corresponding non-deformed array. For the 5 × 5 array configuration, deformations with 100% Uniform distribution and ±100 nm limit values increase the forward-scattering nearly 5 times in average.

As depicted in Fig. 6, the increase rates in the forward-scattered field values (enhancement factors) are different for the single nanowire and arrays. In order to analyse the effect of the number of nanowires to the enhancement factor, Table 1 lists computational results obtained for different arrangements of non-deformed and deformed nanowires. In addition to single nanowires, 2 × 2, 3 × 3, 4 × 4, 5 × 5, 6 × 6, 7 × 7, 8 × 8, 9 × 9, and 10 × 10 arrays, each illuminated by a Hertzian dipole, are considered. Aggressive deformations with 100% Uniform distribution and ±100 nm limit values are applied to generate 5 different deformed samples in each case. It can be observed that the enhancement factor makes a peak with a value of 5.07 for the 4 × 4 array. Then, it becomes smaller when more nanowires are added, with another peak for the 9 × 9 array. The enhancement peaks seem to be related to the cross-sectional electrical size of the array, while no simple analysis is available to predict them without full-wave solutions. Although not shown in Table 1, the enhancement factor further decreases for larger arrays, but always stays greater than 2, which corresponds to the value for infinitely large structures.

## Conclusions

Deformed nanowires as isolated and in array configurations are analyzed using a full-wave simulation environment based on surface formulations and MLFMA. Effects of deformations on scattering properties of nanowires and their arrays are investigated rigorously with respect to the scattering characteristics of perfect structures. Different random distributions, maximum deformation limits, and excitations are considered for a thorough analysis. Based on hundreds of simulations, we identify relatively strong field enhancements in forward-scattering directions for both single nanowires and array configurations.

## Author Contributions

Ö.E. proposed the analysis of deformed nanowires using MLFMA. B.K. designed the experiments. A.Y. did the experiments. Ö.E. and B.K. interpreted the results.

## Figures and Tables

**Table 1:** Forward-scattered field values for non-deformed and deformed nanowires (mean and standard deviation values based on 5 trials), each illuminated by a Hertzian dipole, as well as the corresponding enhancement for different arrangements of nanowires. All deformations are in the [−100, 100] nm range

Geometry	Non-Deformed (V)	Deformed Mean (V)	Deformed Standard Deviation (V)	Enhancement Factor
Single Nanowire	0.752	1.94	0.0656	2.58
2 × 2 Nanowires	3.94	10.6	0.858	2.68
3 × 3 Nanowires	6.56	23.8	1.22	3.63
4 × 4 Nanowires	8.36	42.4	1.63	5.07
5 × 5 Nanowires	13.6	66.0	1.61	4.87
6 × 6 Nanowires	21.0	90.0	1.43	4.29
7 × 7 Nanowires	32.4	119	1.67	3.67
8 × 8 Nanowires	35.8	136	0.952	3.81
9 × 9 Nanowires	34.2	150	4.42	4.38
10 × 10 Nanowires	58.2	176	4.36	3.03
